# Bioaccumulation of metals by algae from acid mine drainage: a case study of Frongoch Mine (UK)

**DOI:** 10.1007/s11356-022-19604-1

**Published:** 2022-03-14

**Authors:** Tianhao Du, Anna Bogush, Paul Edwards, Peter Stanley, Ana T. Lombardi, Luiza C. Campos

**Affiliations:** 1 Department of Civil, Environmental & Geomatic Engineering, Faculty of Engineering, University College London, London, WC1E 6BT UK; 2 Centre for Agroecology, Water and Resilience, Coventry University, Coventry, CV8 3LG UK; 3 Natural Resources Wales, 29 Newport Road, Cardiff, CF24 0TP UK; 4 Botany Department, Federal University of São Carlos, São Carlos, SP 13565-905 Brazil

**Keywords:** Acid mine drainage, Hyperaccumulation, Green algae, Bioaccumulation, Bioindicator, Metals removal

## Abstract

In Frongoch Mine (UK), it is unclear the distribution of metals on indigenous algae and whether these species of algae can accumulate metals. This study aimed to investigate the role of indigenous algae for metal removal from acid mine drainage and understand if metals can be adsorbed on the surface of algae or/and bioaccumulated in algae. A sequential extraction procedure was applied for algae samples collected from acid mine drainage (AMD) water to identify the forms in which metals are found in algae. Concentrations of Fe, Pb, Zn, Cu and Cd were evaluated in the algae and AMD samples were collected in June and October 2019. AMDs samples had a pH value ranging between 3.5 and 6.9 and high concentrations of Zn (351 mg/L) and Pb (4.22 mg/L) that exceeded the water quality standards (Water Framework Directive, 2015). Algae *Ulothrix* sp*.* and *Oedogonium* sp. were the two main species in the Frongoch AMDs. The concentrations of metals in algae ranged from 0.007 to 51 mg/g, and the bioconcentration factor of metals decreased in the following order: Fe >  > Pb >  > Cu > Cd > Zn. It was found that Zn, Cu and Cd were adsorbed onto the surface of and bioaccumulated in the algae, while Pb and Fe were mainly bioaccumulated in the algae. Indigenous algae can be considered as a biogeochemical barrier where metals are accumulating and can be used in bioremediation methods. Also, indigenous algae could be used as a bioindicator to assess water pollution at Frongoch Mine and other similar metal mines.

## Introduction

Acid mine drainage (AMD) has a deleterious impact associated with metalliferous mineral exploitation, leaching potentially toxic metals that can affect the environment (Alpers and Nordstorm 1997; Hudson-Edwards et al. 2011; Nordstrom 2011; Favas et al. 2016; Bogush et al. 2016; Rambabu et al. 2020; Sahoo et al. 2020). Generally, AMD is harmful to the environment nearby and is also thought to affect areas downstream where metals can be transported at some distance (Kumari et al. 2010; Ighalo et al. 2022). For example, AMD can increase river water turbidity due to soil erosion and cause precipitation layering on the riverbed and stream bottom, changing the habitat for organisms (Kumari et al. 2010). Aquatic plants and animals can also be deleteriously affected (Kumari et al. 2010). However, they not only act as the receptor of the contamination but also act as the pathways of metals to humans (Kumari et al. 2010). Thus, metals such as Cd, Pb, Cu and Ni from AMD can be accumulated in the human body through those pathways and cause some diseases (Carolin et al. 2017).

Algae can hyperaccumulate metals such as the non-nutrient elements Pb and Cd (Akcali and Kucuksezgin 2011; Diop et al. 2016), as well as those with a known nutritional function such as Cu, Zn and Fe (Orandi and Lewis 2013; Samal et al. 2020). Studies have shown that algae present in streams receiving AMD can be used as a bioindicator and bioremediation to infer high concentrations of metals and have the capacity to remove metals from within the watercourse (Al-Homaidan et al. 2011; Bwapwa et al. 2017). It has already been confirmed that marine algae like *Ulva* sp. can be used as a bioindicator to provide information about the level of metal concentration in the aquatic environment (Rybak et al. 2012). In addition, green algae *Microspora and Ulothrix* were reported to be used as a bioindicators of acid mine drainage (Equeenuddin et al. 2021). Several studies have reported that algae have the ability to hyperaccumulate Fe, Zn, Cu, Ca, Mg, Pb, Ni and Cd (Akcali and Kucuksezgin 2011; Diop et al. 2016; Equeenuddin et al. 2021)..

The aim of this study was to investigate the role of indigenous algae for metal removal from acid mine drainage and understand if elements can be adsorbed on the surface of algae or/and bioaccumulated in algae in the Zn-Pb Frongoch mining area (UK). A sequential extraction procedure was applied the first time for algae samples collected from AMD water from the Zn-Pb Frongoch mining area (UK) to identify metal speciation in algae. It is proposed that this study will provide a better understanding of the spatial scale of the natural environment that has been affected by AMD and will inform our future remediation methodology for Frongoch Mine.

## Methods

### Study site and samples collection

**Fig. 1:**
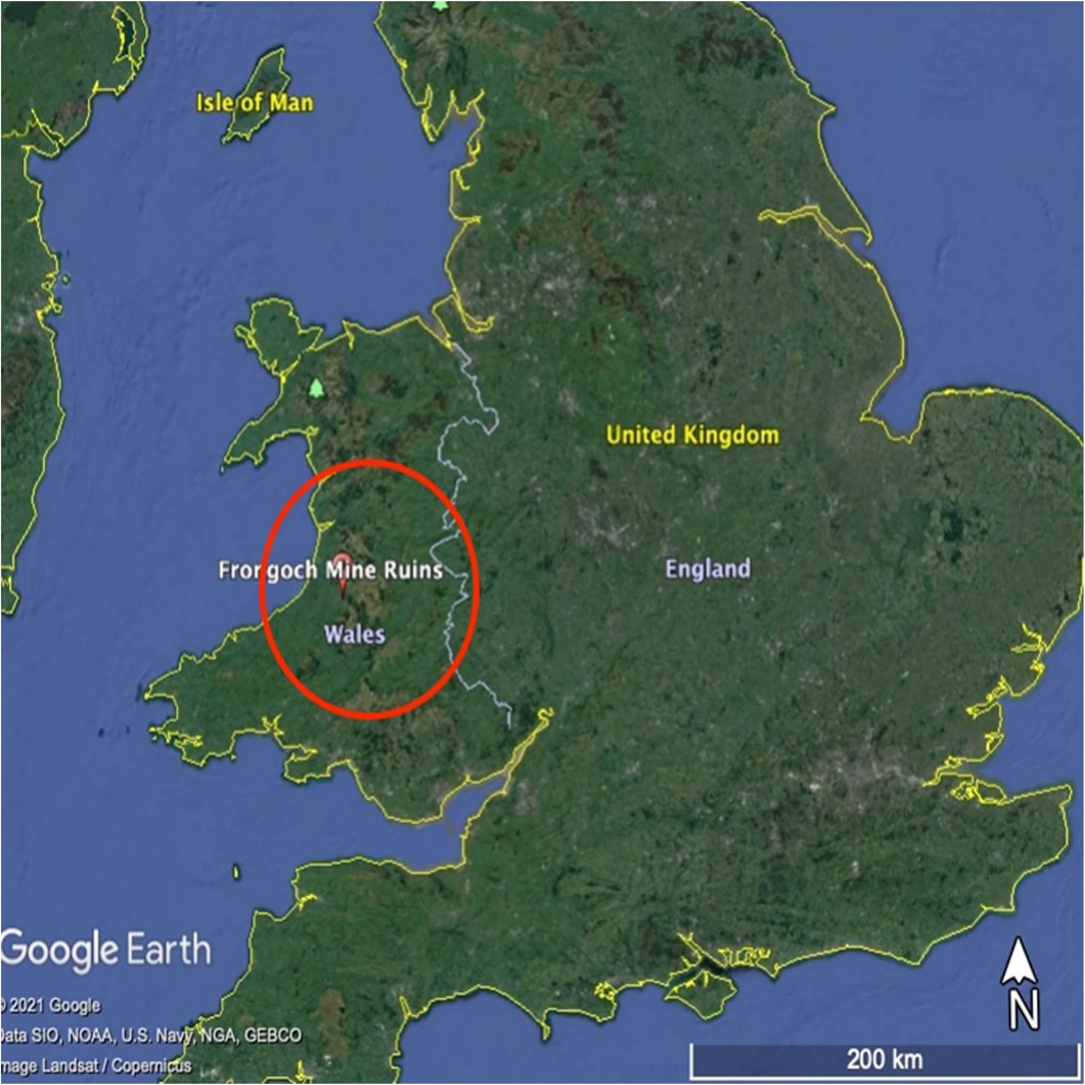
The location of Frongoch Mine in the UK. © Google and Digital Globe (2021)

The study site is the abandoned Pb–Zn Frongoch Mine (Fig. 1), one of the largest metal mines in Mid Wales (Murphy et al. 2015). It is located in a rural area near the village of Pont-rhyd-y-groes, approximately 245 m above ordnance datum and 17 km south–east of Aberystwyth (Edwards et al. 2016). Large scale extraction of Zn and Pb took place in the eighteenth and nineteenth centuries, producing in total 58 K tons of Pb and 50 K tons of Zn (Edwards et al. 2016). Underground mining ceased in 1904, with the large spoil dumps reprocessing from 1917 to 1930. The mine remains the primary contamination source of the Nant Cell and Nant Magwr (tributaries of the River Ystwyth), meaning the water quality does not meet the water quality standards (The Water Framework Directive (Standards and Classification) Directions (England and Wales) 2015).

**Fig. 2:**
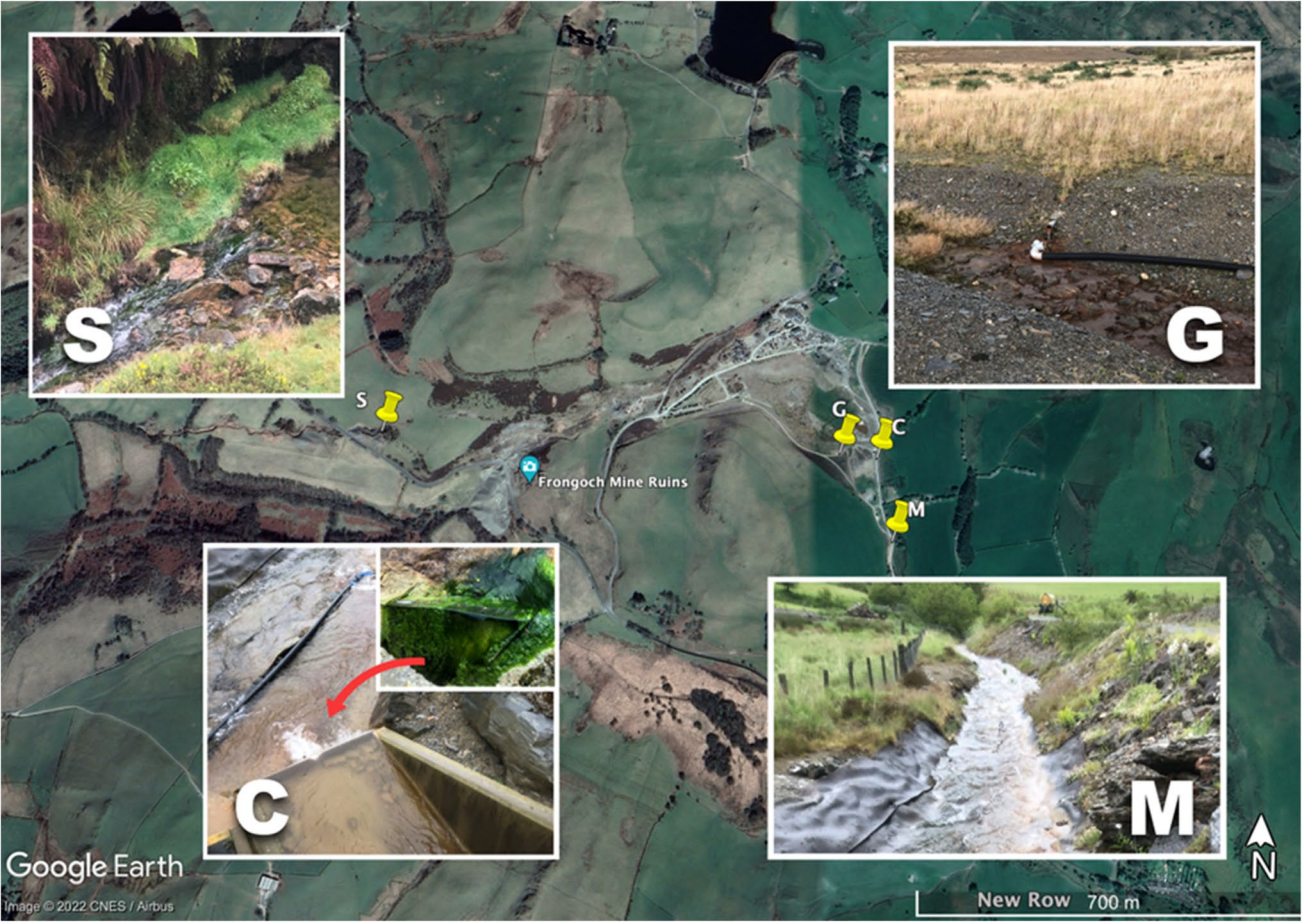
The four sample collection sites in Frongoch Mine. © Google and Digital Globe (2022). Site C is the culvert water from Frongoch tailings; Site M is a mixed stream downstream of Site C; Site G is  the Frongoch groundwater discharged; Site S is a discharge stream from Frongoch Adit

The sampling points are shown in Fig. 2. Site C is the culvert water from Frongoch tailings, and site M is a mixed stream, and culvert water downstream of Site C. Site G is the Frongoch groundwater discharged from a relief drainage pipe beneath the cap. Site S is a discharge stream from Frongoch Adit that passes processed mill tailing heaps prior to joining Nant Gwyn and Nant Cwmnewydion.

AMDs were collected into 500-mL clean plastic bottles, and algae samples from the stream were collected into 25-mL sterile tubes. All the algae samples were filled with AMD water to maximum capacity, avoiding contact with air. After collection, samples were kept in an ice pack bag and transferred to 4 °C fridges (for water samples) and room temperature (for algae) until further analysis (up to 2 h).

### Physical and chemical analysis

Algae samples were stored in the original AMD water. They were examined under an optical microscope and through micrographs obtained using a Zeiss Axio Lab A1 microscope and an AxioCam ERc5s digital camera. The algal strains were identified according to specialised literature (Bicudo and Menezes 2017) and confirmed by an algae specialist from the Federal University of São Carlos. The sequential extraction procedure to identify metal speciation in algae was carried out on the algae samples by two steps of water-leaching (e.g. adsorbed metals on the algae surface) and acid digestion (adapted from (Jaiswar et al. 2015); e.g. bioaccumulated metals in algae). Algae samples (3 g) were dried at 60 °C in the oven for 24 h to ensure all moisture was removed. All the visible sediment particles were removed manually with tweezers before the water-leaching procedures. The water-leaching procedure of dried algae samples included two steps using deionised water with a pH value of around 5.5. In each step, the liquid to solid ratio (L/S) was 10 (5 mL/0.5 g). In the first step, algae samples were transferred to tubes with ultra-pure water and agitated by hand for 30 s to remove the metals attached to the algae surface (water-leachate 1); Then, the algae residue from the first step was mixed with ultra-pure water and placed in a rotating extraction machine for 24 h at 30 rpm to further remove the metals adsorbed on algae surface (water-leachate 2). All the water leachates were centrifuged, filtered, acidified and analysed for metal concentration. Microwave acid digestion was applied to dissolve all the algae residue from two water-leaching steps for metal concentration analysis [adapted from (Jaiswar et al. 2015)]. Ten millilitres of 70% HNO_3_ was added to the algae residue (after the second water-leaching). Then, the microwave was set at the digestion temperature up to 200 °C for 15 min and kept at this temperature for another 15 min. The pH values of the AMD samples and water-leachates were recorded using a pH metre (Mettler Toledo, USA). The AMD samples and water-leachates were filtered through 0.45-µm high-density polyethylene (HDPE) syringe filters (MILLEX® HA) and acidified by ultrapure 70% HNO_3_ to 1% acidity for ICP-OES analysis. The concentrations of metals in the water-leachates and digests were determined by inductively coupled plasma-optical emission spectrometry (ICP-OES) (Varian ICP-OES 720-ES, USA).

### Bioconcentration factor calculation

The bioconcentration factor (BCF) is defined as the ratio between the metal concentration in algae and the concentration of the metals in the AMD sample. BCF provides information about the availability of algae to accumulate metals in its tissue. This was calculated by Eq. (1) (Diop et al. 2016).

1 $$BCF={C}_{a}\times 1000/{C}_{w}$$

where *C*_a_ and *C*_w_ represent the concentration of the metals in algae (mg/g) and the concentration of the metals in the AMD sample (mg/L), respectively.

## Result and discussion

### The pH value of AMD in Frongoch Mine

The pH values of AMD samples from the four sites ranged from 3.5 to 6.9 (Table 1). The lowest pH value was identified in AMD samples from site G. The highest pH values, which were nearly neutral, were from AMD samples of the sites S and M. Comparing with the water quality standards (The Water Framework Directive (Standards and Classification) Directions (England and Wales) 2015), AMD samples from sites G and C are not within the standards of permissible pH = 6.0–9.0.

**Table 1:** The metal concentration of acid mine drainage from the Frongoch Mine (mean value ± SD)

	Metals					
Sample	pH	Zn (mg/L)	Pb (mg/L)	Cd (mg/L)	Cu (mg/L)	Fe (mg/L)
S1	6.19	13.8 ± 0.25	< DL^†^	0.03 ± 0.01	< DL^†^	0.01 ± 0.01
C1	4.81	84.1 ± 0.93	4.22 ± 0.16	0.23 ± 0.04	0.08 ± 0.01	< DL^†^
G1	3.57	314 ± 3.01	2.93 ± 0.13	0.44 ± 0.01	0.01 ± 0.01	0.29 ± 0.01
S2	6.85	15.7 ± 0.32	0.64 ± 0.14	0.03 ± 0.01	0.05 ± 0.01	< DL^†^
C2	4.89	139 ± 1.85	1.74 ± 0.14	0.38 ± 0.01	0.18 ± 0.02	< DL^†^
G2	3.46	351 ± 3.18	3.8 ± 0.174	0.5 ± 0.02	< DL^†^	1.42 ± 0.01
M2	6.59	7.32 ± 0.52	< DL^†^	< DL^†^	0.2 ± 0.01	< DL^†^
Water quality standards	6–9	0.0109	0.014	0.0015	0.001	1

†*DL*, detection limit; *Zn*, 0.0079 mg/L; *Pb*, 0.074 mg/L; *Cd*, 0.0044 mg/L; *Cu*, 0.0039 mg/L; *Fe*, 0.0044 mg/L

The wide range of the pH values among samples was caused by the location of different sample collection points. Site G and C were directly discharged from acid mine drainage pipe and culvert, respectively, so water from these two sites has the lowest pH value, while M is a mix of water from site C and freshwater streams. Thus, the pH value in site M is much higher than that from site G. In terms of water from site S, it just passes around the tailing heap, so the pH of site S was not severely affected by tailings.

Compared with some other mine sites around the world, the pH value of the Frongoch Mine is in the middle level. Nordstrom (2011) reviewed some studies about AMD and concluded that the pH of AMD can range from − 3.5 to 5. In general, most mine sites around the world have a pH ranging from 2 to 5. For example, Ruehl and Hiibel (2020) mentioned Perry Canyon copper mine in the USA has a pH value of 2.81. Grawunder et al. (2014) reported that a pyrite mine in Sweden has a pH value of 3.2, similar to our site’s lowest pH. They also mentioned the pH value of a uranium mine in Germany ranged from 4.4 to 5.6. Likewise, Equeenuddin et al. (2021) also studied algae around cooper mine in central India, and their AMD pH ranged from 4.47 to 5.75.

### Algae microphotograph identification

**Fig. 3:**
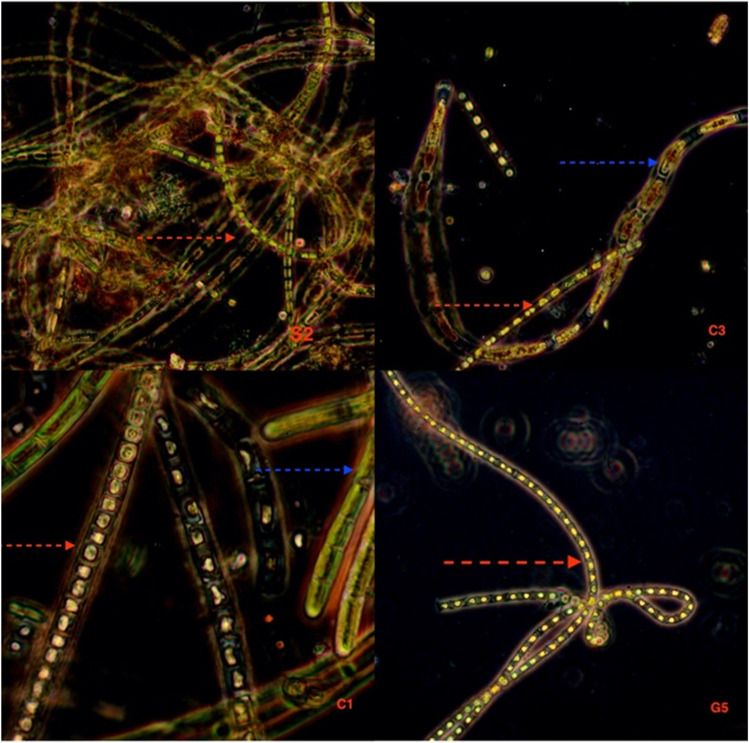
Microphotographs of algae samples. Two types of algae are *Ulothrix* sp. (red arrow) and *Oedogonium* sp. (blue arrow)

The microphotographs of algae (Fig. 3) show two types of algae *Ulothrix* sp. (red arrow) and *Oedogonium* sp. (blue arrow) that were identified in the Frongoch Mine AMDs. Samples C1 and C3 have a collection of these two types of algae, and samples S2 and G5 only have *Ulothrix* sp. Those two species of algae are commonly found in acid mine drainage and have also been found around mine sites by other researchers (Bakatula et al. 2014; Equeenuddin et al. 2021).

### Metal concentrations in the Frongoch Mine AMD

Table 1 shows the metal concentrations in AMD from Frongoch Mine. The highest Zn concentration was observed on site G that exceeded the water quality standards over 32,000 times. Moreover, the Zn concentrations in all other samples from sites C, M and S exceed the water quality standards by 7300–7700 times, 670 times and 1440 times, respectively (Table 1). The highest Pb concentrations were observed at sites C and G, exceeding the water quality standards by 301 and 271 times, respectively. Cd had a relatively low concentration (0.03–0.5 mg/L) in the AMD; however, it exceeded the water quality standards by 333 times at the G site. The Cu concentrations are relatively low and only exceed the water quality standards by 10–200 times at sites G, C and M. However, site G had the highest Fe concentrations, which only exceeded the water quality standards by 1.4 times. The concentrations of Fe on other sites were under the water quality standards. The high concentration of metals, particularly Zn and Pb, was attributed to the AMD water being received from the abandoned Pb–Zn Frongoch Mine. The Pb–Zn mine and the formation of acidic condition may have led to intensive leaching of potential pollutants from metal-bearing waste rock and tailings (Gwenzi et al. 2017). The metal concentration was slightly higher in the autumn sampling (S2, C2 and G2) period than in summer (S1, C1 and G1). Inter-seasonal variation of metal concentration in the AMD was also observed by Oh and Yoon (2013). They reported that the metal concentration in AMD in summer samples was significantly higher than that in spring samples. Changes in some seasonal parameters such as the amount of precipitation, temperature and runoff may partially explain the changes in concentration of metals (Mondol et al. 2011; Wijngaard et al. 2017).

### Metal concentration and speciation in algae from the Frongoch Mine AMD

**Fig. 4:**
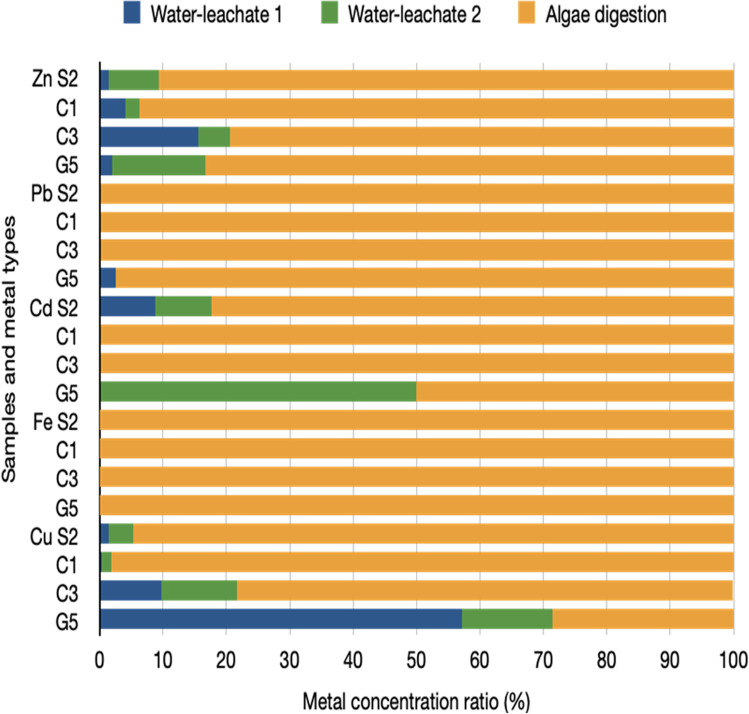
Metal concentration ratio (%) of sequential extraction of metals from algae

Table 2 and Fig. 4 present the results of sequential extraction of Zn, Pb, Fe, Cd and Cu from algae that grow in the Frongoch AMDs. The metal concentrations in algae from AMDs of the Frongoch Mine varied: Fe, 6.3–5 mg/g; Zn, 2.5–19.3 mg/g; Pb, 2.1–32.3 mg/g; Cu, 0.007–1.1. mg/g; Cd, 0.008–0.036 mg/g (Table 2). In general, the total accumulation of metals from algae decreased in the following order: Fe > Pb > Zn > Cd > Cu.

**Table 2:** Sequential extraction of metals from algae living in the Frongoch AMDs

Metals	Leachates from sequential extraction	Metals concentration mg/g			
		S2	C3	C1	G5
Zn	Water-leachate 1	0.142 + / − 0.001	0.046 + / − 0.005	0.079 + / − 0.003	0.052 ± 0.001
	Water-leachate 2	0.786 + / − 0.007	0.148 + / − 0.003	0.436 + / − 0.005	0.371 ± 0.011
	Algae digestion after washing	8.96 + / − 0.054	2.35 + / − 0.033	18.1 + / − 0.162	2.11 ± 0.012
	Sum	9.888 ± 0.062	2.958 ± 0.041	19.327 ± 0.17	2.533 ± 0.024
Pb	Water-leachate 1	< DL†	< DL†	< DL†	0.051 ± 0.034
	Water-leachate 2	0.057 + / − 0.039	< DL†	< DL†	< DL†
	Algae digestion after washing	28.8 + / − 0.287	4.39 + / − 0.096	32.3 + / − 0.358	2.0 ± 0.053
	Sum	28.86 ± 0.326	4.39 ± 0.096	32.3 ± 0.358	2.051 ± 0.087
Cd	Water-leachate 1	0.003 + / − 0.002	< DL†	< DL†	< DL†
	Water-leachate 2	0.003 + / − 0.001	< DL†	< DL†	0.003 ± 0.002
	Algae digestion after washing	0.028 + / − 0.002	0.016 + / − 0.006	0.036 + / − 0.003	0.004 ± 0.002
	Sum	0.034 ± 0.004	0.016 ± 0.006	0.036 ± 0.003	0.007 ± 0.004
Fe	Water-leachate 1	0.002 + / − 0.001	< DL†	0.001 + / − 0.001	0.002 ± 0.001
	Water-leachate 2	0.009 + / − 0.003	0.003 + / − 0.001	0.002 + / − 0.001	0.008 ± 0.001
	Algae digestion after washing	28.9 + / − 0.166	6.34 + / − 0.058	51.2 + / − 0.027	47.8 ± 0.034
	Sum	28.911 ± 0.17	6.343 ± 0.059	51.2048 ± 0.029	47.81 ± 0.036
Cu	Water-leachate 1	0.011 + / − 0.001	0.009 + / − 0.001	0.004 + / − 0.001	0.004 ± 0.001
	Water-leachate 2	0.03 + / − 0.001	0.003 + / − 0.001	0.004 + / − 0.001	0.001 ± 0.001
	Algae digestion after washing	0.738 + / − 0.002	0.072 + / − 0.002	1.09 + / − 0.006	0.002 ± 0.002
	Sum	0.738 ± 0.004	0.092 ± 0.004	1.111 ± 0.008	0.007 ± 0.004

†*DL*, detection limit; *Zn*, 0.0079 mg/L; *Pb*, 0.074 mg/L; *Cd*, 0.0044 mg/L; *Cu*, 0.0039 mg/L; *Fe*, 0.0044 mg/L

The water-leachate 1 and water-leachate 2 had a very low Fe concentration compared with the acid digest of algae (Table 2 and Fig. 3). This indicated that the adsorbed Fe on the algae surface is very low, and Fe was mainly accumulated inside algae by absorption. Oberholster et al. (2014) analysed three species of algae collected from AMD sites in temperate climates and found that Fe had the highest concentration in *Microspora tumidula*, followed by *Oedogonium crassum* and *Klebsormidium klebsii*. A study in Eastern Aegean of algae from Dardanelles contaminated coastal area also reported that Fe had the highest concentration in algae (Akcali and Kucuksezgin 2011). Fe accumulation by algae is known to be important in various metabolic and photosynthesis phases. It binds to the redox-active metal (a cofactor) found in proteins, which functions as a protective mechanism for the survival of the algae (Al-Shwafi and Rushdi 2008; Oberholster et al. 2014). Algae is also known to hyperaccumulate Fe captured in algal biomass (Akcali and Kucuksezgin 2011).

The high concentrations of Zn (79.4–90.6%) in algae were found in the algae acid digest fraction, which indicates that Zn is mainly accumulated by algae. Zn is also an essential micronutrient to promote algae growth but only has positive effects at low concentrations. High Zn concentration in algae can adversely influence the physical and biochemical processes of algae growth (Trzcińska and Pawlik-Skowrońska 2013). Also, some parts of Zn can be adsorbed on the surface of algae (6.4–20.6%) (Fig. 2). In general, the percentages of surface-accumulated Zn in algae collected in summer (C1, 6.4% and S2, 9.4%) were less than for those collected in autumn (C3, 20.6% and G5, 16.7%), but all these ranges are consistent with Knauer et al. (1997), who reported results of 5–80% of Zn accumulation in *Scenedesmus subspicatus* and *Chlamydomonas reinhardtii*. Similarly, a relatively high amount of Cu was also adsorbed on the surface of algae (1.9–71.4%). Knauer et al. (1997) also reported that approximately 20% of Cu was located on the algae surface, and most Cu (approximate 80%) was absorbed intracellularly. In our study, except for G5 (surface Cu percentage 71%), the rest of the three samples (surface Cu percentage 1.9–22%) is in agreement with the results reported by Knauer et al. (1997). A small amount of Cu accumulation is caused by Cu accumulation on algae surface reaching a saturation level when the background water Cu^2+^ ion is > 10^−9^ M (6.4 × 10^−6^ mg/L) (Wang and Dei 2006). As observed with Zn, the percentage of Cu on summer collected algae surface (C1, 1.98% and S2, 5.26%) was lower than that on autumn collected algae surface (C3, 21.74% and G5, 71.3%).

Pb is, however, mainly accumulated inside the algae, as shown by the high Pb content measured in the algae acid digest fraction (> 97.5%). In these four samples, C1 and C3 had 100% Pb concentration in algae, and no Pb was detected in water-leachates 1 and 2, which means all the Pb was accumulated inside the algae, indicating that *Oedogonium* sp*.* has a strong ability in absorbing Pb internally. Several studies (Halder 2014; Bwapwa et al. 2017) also agreed with the Pb bioaccumulation capacity by *Oedogonium*. While Cu and Fe are essential elements for algae, Pb is considered a toxic element (Shanab et al. 2012). The accumulation of Pb in algae may be because algae have evolved to have different metal resistance, which is driven by edaphic conditions (Trzcińska and Pawlik-Skowrońska 2013). Similarly, Cd was also rarely detected in C1 and C3 water leachates. Compared with Pb, however, Cd also had a relatively low concentration inside the algae (0.004–0.036 mg/g). Thus, the total amount of Cd accumulated by algae was lower than the other metals (0.007–0.036 mg/g). The low amount of Cd uptake by algae may be because of (1) the low Cd concentration (0.08–0.5 mg/L) in Frongoch Mine AMD water and (2) high Zn concentration may inhibit Cd uptake by algae (Töpperwien et al. 2007). When the concentration of Zn to Cd ratio is over 14, the Cd accumulation shows a decreasing trend and the cellular Zn increasing (Töpperwien et al. 2007). In this study, the concentration of Zn to Cd ratio ranges from 365 to 702, which is over 14. Thus, the Zn may compete with Cd in accumulation and inhibit Cd uptake (Töpperwien et al. 2007). Based on the discussion above, two types of algae collected from the Frongoch Mine *Ulothrix* sp. and *Oedogonium* sp. were proved to have potential in metal removal from AMD. Some studies also agree with our findings and reported *Ulothrix* sp. as having significant capacity in metals (i.e. Fe, Cd, Cr, Pb, Zn and Cu) removal from AMD (Das et al. 2009; Monteiro et al. 2012; Orandi et al. 2012; Halder 2014; Oberholster et al. 2014).

### The bioconcentration factor of algae

The BCF of algae samples is given in Table 3. The results indicate that the bioaccumulation of metals in algae has a wide range. The BCF of different metals on average decreased in order: Fe >  > Pb >  > Cu > Cd > Zn. Fe, Cu and Zn are biologically essential elements that participate in the different physiological processes in algae. Therefore, Fe had the highest concentration and bioaccumulation ratio in our research, which agrees with the study of Akcali and Kucuksezgin (2011). Although Zn is also a biologically required element, its bioaccumulation factor was the lowest, which is lower than 1. This was probably because Zn had the highest concentration in AMDs and caused the BCF value to be low. Interestingly, BCFs for Pb were high for the samples C1, C3 and S2, with the highest value of 4509 for the sample S2 (Table 3). Pb is an abiogenic element and is not essential for algae. However, Pb can be passively absorbed by algae, and this might be caused by a resistance mechanism, which can make algae evolve chemical speciation for toxic and nonessential metals (Hamidian et al. 2016).

**Table 3:** The biocommunication factor of metals in algae

	Fe	Pb	Zn	Cd	Cu
C1	11637454^‡^	7654	230	122	13887
C3	1441590^‡^	2523	21	421	511
S2	6570681^‡^	4509	630	933	14760
G5	33669	5397	722	14	1794^‡^

‡Calculation based on detection limit

In terms of algae samples, C1 (*Ulothrix* sp. and *Oedogonium* sp.) had the highest capacity to accumulate metals, followed with S2 (*Ulothrix* sp.). Compared with C1 and S2, C3 (*Ulothrix* sp. and *Oedogonium* sp.) and G5 (*Ulothrix* sp.) have a relatively low capacity to accumulate metals. This has apparent seasonal change, particularly compared to the same sampling point samples C1 (collected in summer) and C3 (collected in autumn). The result showed that samples collected in summer had more metals accumulated. Similar results were reported by Yozukmaz et al. (2018), where authors described that both metal concentration and BCF of algae reached the highest level in the summer time and decreased in the autumn. Another study, however, reported increasing metal concentration and accumulation from autumn to winter, followed by a decreasing trend in spring (Bwapwa et al. 2017). The changes in seasons and different results in several studies are probably because of environmental parameters such as pH, salinity, temperature and presence of other elements and different algae communities/species (Akcali and Kucuksezgin 2011). In addition, algae growth rate and metabolic dynamic can also affect the accumulation and concentration of the metal in the algae body; for example, metal concentrations in algae usually decrease during the growing period (Akcali and Kucuksezgin 2011). Another important factor is anthropogenic activity, such as tourism and agriculture, which significantly affect various sites and seasons. In some places, a noticeable increase in the population during spring and summer affects the metal concentration (Yozukmaz et al. 2018).

## Conclusion

This study evaluated AMDs from the abandoned Zn-Pb Frongoch Mine. It confirmed that AMDs had a pH range between 3.5 and 6.9 and high concentrations of Zn and Pb that exceeded the environmental quality standards. The metal concentrations in AMDs are high to low as Zn >  > Pb > Cd > Fe > Cu. *Ulothrix* sp. and *Oedogonium* sp., both characterised as filamentous green algae, were the two most commonly observed species in the Frongoch Mine AMDs. It was found that Zn, Cu and Cd were adsorbed on the surface of the algae and bioaccumulated in the algae, while Pb and Fe were mainly bioaccumulated in the algae. BCF results indicated that Fe had the highest accumulation ratio in algae, followed by Pb, Cu, Cd and Zn. Algae can be considered a biogeochemical barrier where metals are accumulating. Also, indigenous algae could be used as a bioindicator for assessing water pollution at the Frongoch Mine and other similar metal mines since algae have an excellent capacity for metal accumulation.

Further study may focus on examining the statistical relationship between different metals accumulation and background water from other sites to establish the detailed pattern of metal accumulation by algae in this area. Factors also causing seasonal changes should be identified by further sampling and analysis.

## Data Availability

The datasets used and analysed during the current study are available from the corresponding author on reasonable request.
